# Similar stress repartition for a standard uncemented collared femoral stem versus a shortened collared femoral stem

**DOI:** 10.1051/sicotj/2021061

**Published:** 2021-11-19

**Authors:** Cécile Batailler, Jobe Shatrov, Axel Schmidt, Elvire Servien, Jean Marc Puch, Sébastien Lustig

**Affiliations:** 1 Department of Orthopaedic surgery and Sports Medicine, Croix-Rousse Hospital, Lyon University Hospital 103 grande rue de la Croix Rousse 69004 Lyon France; 2 Univ Lyon, Claude Bernard Lyon 1 University, IFSTTAR, LBMC UMR_T9406 69622 Lyon France; 3 Sydney Orthopaedic Research Institute, University of Notre Dame Australia, Hornsby and Ku-Ring Hospital NSW 2067 Sydney Australia; 4 LIBM – EA 7424, Interuniversity Laboratory of Biology of Mobility, Claude Bernard Lyon 1 University 69003 Lyon France; 5 Department of Orthopaedic Surgery, Clinique Saint-Georges 2 Av. de Rimiez 06105 Nice France

**Keywords:** Total hip arthroplasty, Short stem, Uncemented stem, Finite element analysis, Stress repartition

## Abstract

*Introduction*: The design of uncemented femoral stems for use in total hip arthroplasty has evolved. Several uncemented short stems have been developed with different bone fixations, shapes, or stem lengths. The literature analyzing the biomechanical performance of short to standard stem lengths is limited. The aim was to compare the stress repartition on a standard uncemented stem and a shortened uncemented femoral stem with the same design features. *Material and methods*: This finite element analysis assessed the stress repartition on two femoral components with the same design (uncemented, collared, proximal trapezoidal cross-section, and a tapered quadrangular distal stem) but with two different lengths. The shortened stem was shorter by 40 mm compared to the standard stem. The stress repartition was analysed according to the Von Mises criterion. *Results*: The stress repartition was similar for the standard and shorter stem without significant difference (*p* = 0.94). The mean Von Mises stress was 58.1 MPa [0.2; 154.1] for the standard stem and 57.2 MPa [0.03; 160.2] for the short stem. The distal part of the standard stem, which was removed in the short stem, had mean stress of 3.7 MPa [0.2; 7.0]. *Conclusion*: The finite element analysis found similar stress repartitions between a standard uncemented collared stem and a short, collared stem with the same design. A clinical study assessing the clinical outcomes and the bone remodelling with a collared short stem would be interesting to confirm these first promising results.

## Introduction

In the 20th century, total hip arthroplasty (THA) has emerged as a procedure that offers patients one of the greatest improvements in quality of life considering all surgical interventions [1]. A number of studies have reported excellent survivorship with standard uncemented stem types at 20 years follow-up [2]. Several engineering modifications to optimise bone fixation and a preserve bone stock in uncemented THA, including implant shape, surface coating, and modularity, have been developed. Recently, uncemented short-stem femoral components have gained increasing interest in THA. Their proposed benefits over standard length stems include preservation of bone stock in young patients with a risk of several THA revisions over their lifetime [3], minimally-invasive surgery [4], reduced risk of proximal stress shielding [5, 6], reduced thigh pain from diaphyseal loading with longer stems and decreased bone loss around the lesser trochanter due to diaphyseal fixation of longer uncemented stems [7]. Several studies have reported good implant survivorship at short to mid-term follow-up using short-stem uncemented femoral components [8–11].

The precise definition of short-stem prosthesis remains debated, and their design features are heterogeneous. Loading forces depend on the specific geometry of the stem [12]. Recently, Drosos and Touzopoulos classified short-stem designs according to the fixation location, based either on the column or trochanteric region [13]. While the intention of improved biomechanics has been enticing, some short-stem designs have been associated with poor alignment [14–16] and primary instability [17]. Type IVb stems of Drosos classification, which have a shortened femoral component that ends at the meta-diaphyseal junction, aim to achieve a compromise between better proximal fixation and stress repartition on the metaphysis while having a length that avoids misalignment. However, to date, no finite element or biomechanical analysis that assesses the stress repartition comparing a similarly standard stem to the same designed shortened stem has been reported in the literature.

The hypothesis of this study was that the stress repartitions were similar between two similarly designed uncemented collared stems despite two different lengths: a standard stem and a shortened stem. The aim was to compare the stress repartition on a standard uncemented stem compared to a shortened uncemented femoral stem with the same design, using a finite element analysis.

## Material and method

This study was a finite element analysis of two types of uncemented femoral stems. The model was created by the ProMechanica software (Bright Hub, Albany, NY, version 23.3). It reproduced the implantation of a primary femoral stem in a bloc designed to match the physiological behaviour of cortical and cancellous bone in the proximal femur.

### Implant

**Figure 1:**
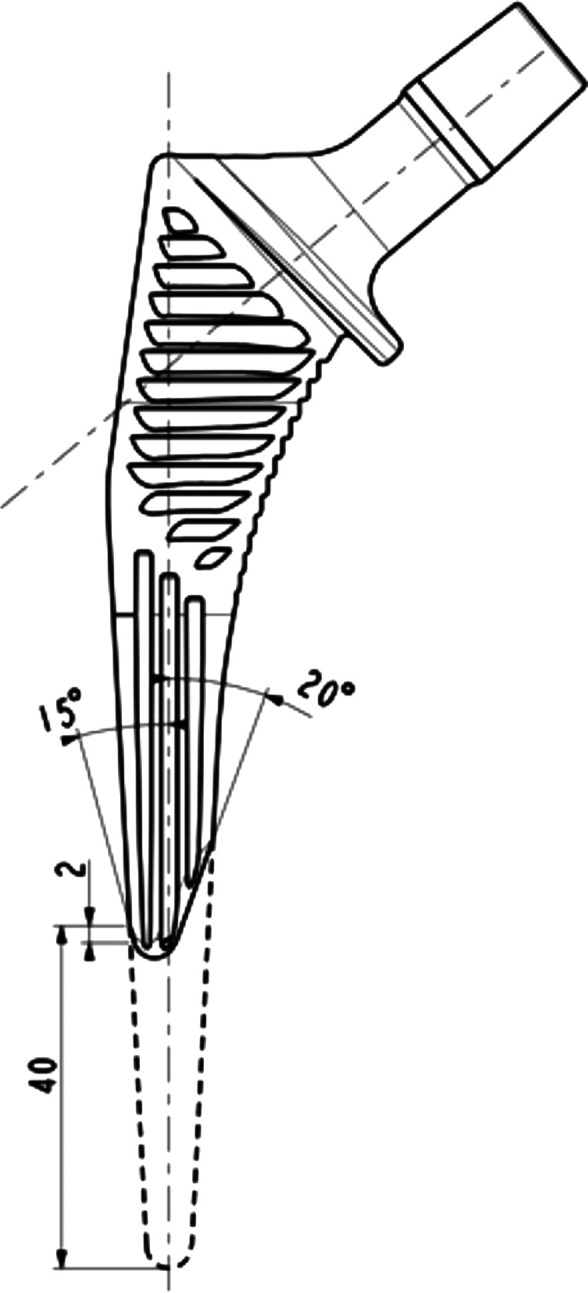
Design of the Targos stem size 9 with the shape of the standard stem and the shortened stem.

Two different femoral stems were analyzed, Targos (Lepine^®^, Genay, France) and a shortened stem, Targos Mini (Lepine^®^, Genay, France). This prosthesis gains cementless fixation and has a proximal bone collar. The design includes a proximal trapezoidal cross-section intended to resist axial/torsional stresses and promote initial stability, and a tapered distal stem that is quadrangular provides a decreasing stiffness gradient to reduce the elastic mismatch between the prosthesis and bone. Each implant has a nonporous fully hydroxyapatite coating on a forged titanium alloy stem. The only difference between the two stem designs was their respective lengths, with the shortened stem being shorter by 40 mm compared to the standard stem. The metaphyseal part was the same between these two implants (Figure 1).

### Stress test

**Figure 2:**
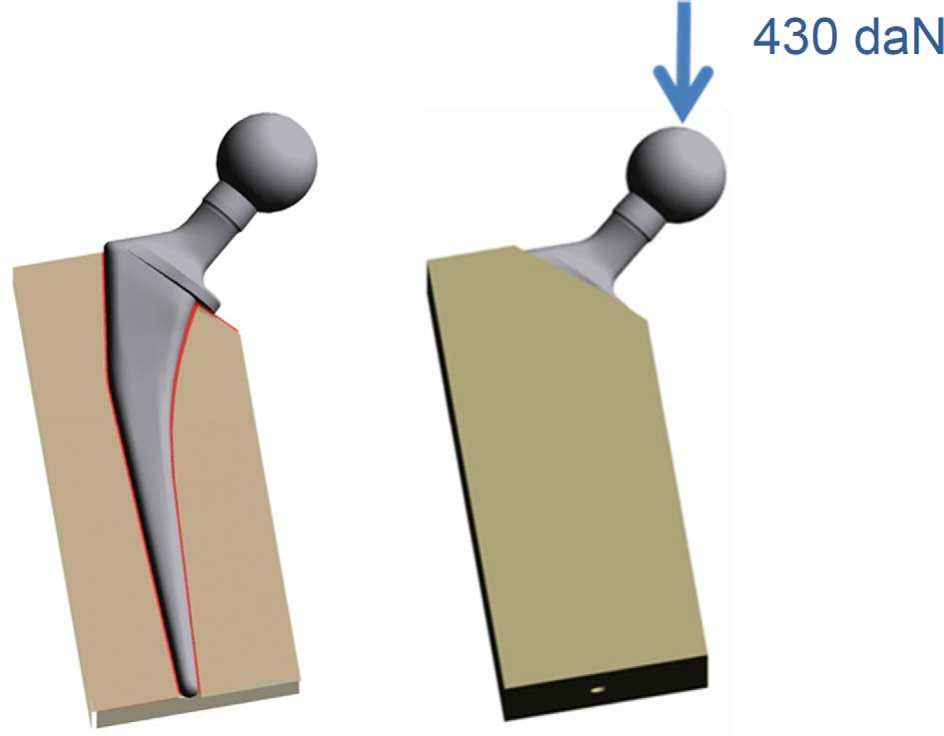
Model principle and loading of the stem which is introduced in a bloc with the same mechanical properties as cortical bone.

The two stems were implanted in a bloc with the same mechanical properties as cortical bone. A 0.1 mm clearance was created around the stem to reproduce the cancellous bone like in-vivo conditions. To reproduce in-vivo forces, the stem was positioned in the femoral shaft without varus or valgus malalignment. The collar was entirely supported by the bone model to create complete contact between the collar and calcar region. A compressive load of 430 daN was applied vertically on the femoral head (Figure 2). The stress repartition was analyzed by the Von Mises criterion. The stress repartition was noted along the external line of the stem. The Von mises stress was reported according to the localizations on the stem and the distance from the distal end of the stem. The same analyses were performed for the two types of stems.

### Statistics

Statistical analysis was performed using the XL STAT software (Addinsoft, Paris, France). Continuous variables were averaged and reported with minimum and maximum values. Significant differences were calculated using Student’s *t*-test for continuous variables. A *p*-value < 0.05 was considered statistically significant.

## Results

**Figure 3:**
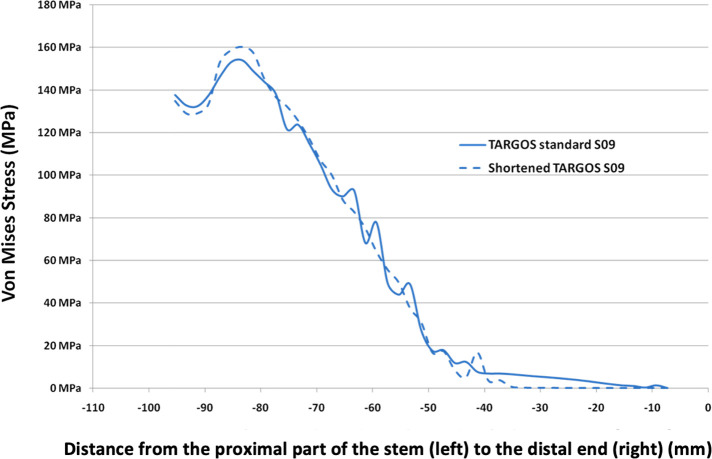
Comparison of Von Mises stress values on the external line of the stem with a standard stem and a shortened stem (−40 mm).

The stress repartitions were similar for the standard stem and the shortened stem according to the distance from the distal end of the stem, without any significant difference (*p* = 0.94) (Figure 3). The overall Von Mises stress repartitions on the femoral stems were constant regardless of stem length.

**Figure 4:**
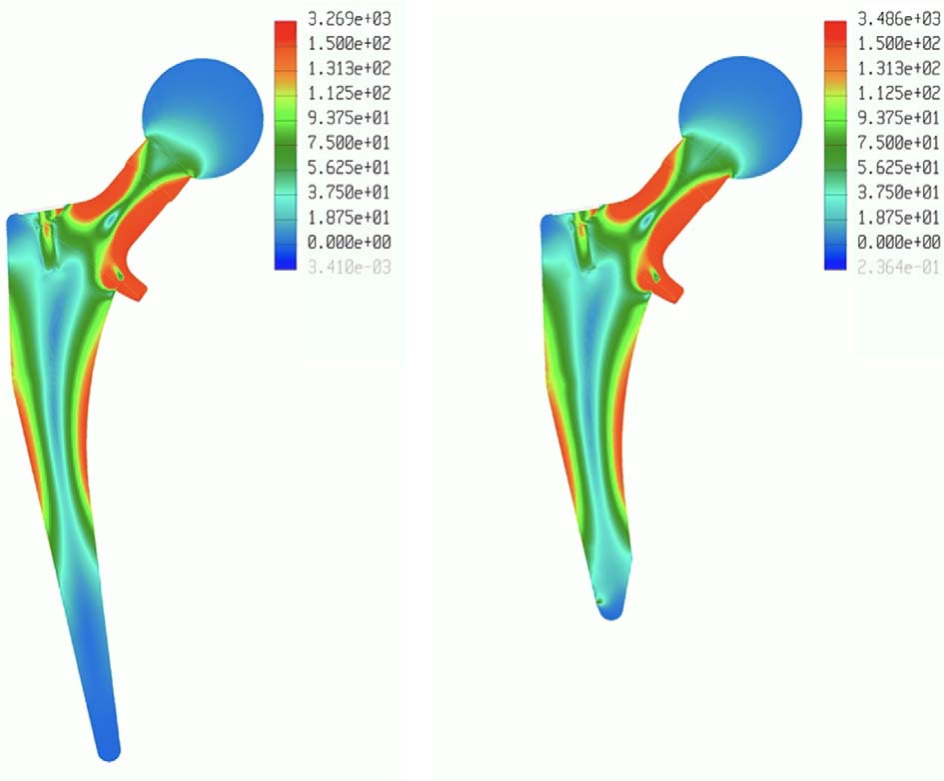
Diagrams representing the Von Mises stress repartition for a standard collared stem and a shortened collared stem.

Stress distributions were similar between both stems, with the highest values being localized at the proximal part of the stem, including the metaphyseal part, femoral neck, and collar. Peak stress values were not higher in the shortened stem (Figure 4). The distal portion of the standard stem had stress inferior to 10 MPa compared to the collar, which had to stress superior to 150 MPa. The mean Von Mises stress was 58.1 MPa [0.2; 154.1] for the standard stem and 57.2 MPa [0.03; 160.2] for the shortened stem. The distal part of the standard stem, which was removed in the shortened stem, had mean stress of 3.7 MPa [0.2; 7.0].

## Discussion

The main finding of this study was that stress repartition did not differ between two similar designed uncemented collared stems despite different stem lengths (standard and shortened type). Furthermore, the distal part of the stem exhibited very low-stress values. The main areas of stress were localized to the proximal portion of the stem, including the metaphyseal region, femoral neck, and collar.

Short-stem femoral components have been designed to reduce proximal stress shielding, preserve bone stock, and decrease the elastic mismatch at the stem-bone interface compared to standard stem lengths. Finite element analyses have previously been used to compare metaphyseal short to standard stems, with several studies reporting a more physiological stress distribution proximally with metaphyseal stem designs [5, 12]. Yan et al. compared a metaphyseal short to a standard hip stem, reporting a reduction of the cortical stress in the proximal femur in both designs [18]. However, the shorter stem was characterized by a metaphyseal load transfer, the standard stem featured a combination of metaphyseal and more diaphyseal load transfer, thus concluding the short stem had better restoration of the native cortical stress compared to a standard stem. Interpreting differences between short and standard uncemented stems previously, however, has been difficult due to major design differences such as the fixation area and the stem shape. The findings of the present study are interesting as the only difference between prosthesis designs was the length, controlling for aforementioned potential confounders. Thus, the biomechanical influence of stem length in this design can be fully understood.

The effect of stem length in different femoral prosthesis has been examined previously. A cadaveric study assessing a standard, cementless, collarless stem that was shortened by 40–50 mm found no effect on stem stability but normalized the load distribution in the lower metaphysis and upper diaphysis [19]. The authors concluded that the distal 30–50 mm of the original stem was responsible for stress shielding seen in the lower metaphysis and upper diaphysis. This observation was not observed in the present study and is likely due to the presence of a collar in this prosthesis, although it could also be due to the design differences between the studies. Proximal stress shielding secondary to a distal fixation of the stem has already been described in the literature [20] and is one of the advantages of short stems. Mechanical in vitro studies on short stem micromotion is limited mainly to metaphyseal short stems. Two systematic reviews of comparative studies comparing prosthesis designs of short stems have found that periprosthetic bone remodelling was also present in short stems (mainly proximally), and a distinctively different pattern between the types of design [12, 21]. The osteointegration and the survivorship of short stem femoral implants are currently satisfying, with low revision rates reported (median revision rate of 4.8% after 10 years) [9, 22, 23].

In this present study, the stems had a collar. Few short metaphyseal stems have been designed with the use of a collar. Calcar resorption or osteolysis has been described with the use of collared stems previously [24, 25]. However, countering this are several studies that have reported less stem subsidence and early periprosthetic fracture with the use of a collared prosthesis [26–28]. The advantages of a collar with a shortened stem have not been assessed in the literature. However, this study did not find a significant difference in the stress repartition on the calcar with a standard stem or a shortened stem. A clinical study assessing the clinical outcomes and the bone remodelling with a collared shortened stem would be interesting to confirm these first promising results.

There were several limitations in this study. Firstly, this study has assessed only one type of short stem with a specific design, particularly a collar. Therefore, the results of this study may only be extrapolated for a similarly designed prosthesis. Second, this was a finite element analysis with bone modelization. While this form of analysis has been validated previously in THA, the stress repartition can vary in-vivo according to individual bone quality and morphology. Finally, loading of the stem was vertical and may have only partially reproduced the constraints on a THA in-vivo.

Despite these limitations, which are an inherent design issue with in vitro analysis, to our knowledge, this is the first study comparing stress repartition of a shortened collared stem and a standard stem with the same design, with the only difference in design being the length of the stem.

## Conclusion

The finite element analysis found similar stress repartitions between a standard uncemented collared stem and a shortened collared stem with the same prosthesis design. A clinical study with these implants is necessary to confirm these initial encouraging results.

## Conflict of interest

CB: Institutional research support to Lepine. Grant from SoFCOT (Société Francaise de Chirurgie Orthopédique et Traumatologique).

JS, AS: declare that they have no conflict of interest.

ES: institutional research support from Corin.

JMP: Royalties from Lepine.

SL: Consultant for Stryker, Smith and Nephew, Heraeus, Depuy Synthes. Institutional research support to Lepine and Amplitude. Editorial Board for Journal of Bone and Joint Surgery (Am).

## Funding

This research did not receive any specific funding.

## Ethical approval

Ethical approval was not required.

## Informed consent

This article does not contain any studies involving human subjects.

## Authors’ contributions

CB: Study design, statistical analysis, literature review, and manuscript writing.

JS: Literature review and manuscript editing.

AS: Literature review and manuscript editing.

ES: Study design and manuscript editing.

JMP: Study design and manuscript editing.

SL: Study design, manuscript editing, and supervisor.
